# Recommendations for the screening of paediatric latent tuberculosis infection in indigenous communities: a systematic review of screening strategies among high-risk groups in low-incidence countries

**DOI:** 10.1186/s12889-018-5886-7

**Published:** 2018-08-06

**Authors:** Lena Faust, Anne McCarthy, Yoko Schreiber

**Affiliations:** 1 Faculty of Health Sciences, University of Ottawa, Ottawa, Canada; 2 Department of Medicine, University of Ottawa, Ottawa, Canada; 3 Ottawa Hospital Research Institute, Ottawa, Canada

**Keywords:** Latent tuberculosis infection, Indigenous communities, Targeted screening

## Abstract

**Background:**

Tuberculosis (TB) continues to be a global public health concern. Due to the presence of multiple risk factors such as poor housing conditions and food insecurity in Canadian Indigenous communities, this population is at particularly high risk of TB infection. Given the challenges of screening for latent TB infection (LTBI) in remote communities, a synthesis of the existing literature regarding current screening strategies among high-risk groups in low-incidence countries is warranted, in order to provide an evidence base for the optimization of paediatric LTBI screening practices in the Canadian Indigenous context.

**Methods:**

A literature search of the Embase and Medline databases was conducted, and studies pertaining the evaluation of screening strategies or screening tools for LTBI in paediatric high-risk groups in low-incidence countries were included. Studies focusing on LTBI screening in Indigenous communities were also included, regardless of whether they focused on a paediatric population. Their results were summarized and discussed in the context of their relevance to screening strategies suitable to the Canadian Indigenous setting. Grey literature sources such as government reports or policy briefs were also consulted.

**Results:**

The initial literature search returned 327 studies, with 266 being excluded after abstract screening, and 36 studies being included in the final review (original research studies: *n* = 25, review papers or policy recommendations: *n* = 11). In the examined studies, case identification and cost-effectiveness of universal screening were low in low-incidence countries. Therefore, studies generally recommended targeted screening of high-risk groups in low-incidence countries, however, there remains a lack of consensus regarding cut-offs for the incidence-based screening of high-risk communities, as well as regarding the utility and prioritization of individual risk-factor-based screening of high-risk groups. The utility of the TST compared to IGRAs for LTBI detection in the pediatric population also remains contested.

**Conclusions:**

Relevant strategies for targeted screening in the Canadian Indigenous context include community-level incidence-based screening (screening based on geographic location within high-incidence communities), as well as individual risk-factor-based screening, taking into account pertinent risk factors in Indigenous settings, such as poor housing conditions, malnutrition, contact with an active case, or the presence of relevant co-morbidities, such as renal disease.

## Background

Tuberculosis (TB) is a bacterial infectious disease caused by *Mycobacterium tuberculosis*, and continues to be a major global public health concern, with a global incidence of 10.4 million cases and an estimated 1.4 million deaths worldwide being attributed to TB in 2015. Populations particularly at risk of TB infection include immune-compromised individuals such as those with HIV, whilst further significant risk factors for TB acquisition include overcrowded housing, insufficient access to sanitation, and inadequate nutrition [1, 2]. These risk factors highlight the nature of TB as a disease that largely follows a social gradient, therefore underlining the need to consider the social determinants of health and the needs of specific high-risk populations in the design and implementation of strategies for the management of the disease. With this in mind, Indigenous communities must be given particular consideration in TB management efforts [3], due to the confluence of factors such as poor housing conditions [4], food insecurity, and a higher prevalence of co-morbidities in these communities [5].

Indigenous communities in Canada represent a high-risk population for TB infection, with an incidence of 20.6/100,000 in 2014, compared to an incidence of only 0.6/100,000 in the non-Indigenous Canadian-born population [6]. Apart from working towards addressing the underlying socioeconomic conditions that perpetuate the transmission of TB within these communities and contribute to suboptimal health outcomes, the early identification of latent TB infection (LTBI) is crucial in reducing the emergence of new active TB cases and subsequently reducing the transmission of the disease [7]. To this end, the World Health Organization (WHO) recommends targeted screening for LTBI among high-risk individuals (such as those with HIV or those in contact with individuals with active TB) [3]. More specifically in the Canadian context, the *Canadian Tuberculosis Standards* [5] identify the Indigenous population as a high-risk group for TB, and therefore recommend targeted screening for LTBI in this group, although the specific subgroups in which targeted screening is most warranted varies by community, due to differences in the prevalence of the disease across communities [5, 7].

Notably, Indigenous communities in Canada experience a higher prevalence of TB among children than the general population, [5] as is also the case for Indigenous children in other geographic locations, such as among the Inuit of Greenland, [8] and Aboriginal children in Australia [9]. Given that LTBI in young children has also been found to be more likely to progress to active TB, [10–12] the paediatric Indigenous population represents an important priority group for LTBI screening in Canada.

The Tuberculin Skin Test (TST) and Interferon Gamma Release Assays (IGRAs) are the two currently used screening tools for LTBI [2, 13]. The TST is administered via injection into the skin of the arm, and is interpreted by considering the extent of the resulting cell-mediated immune response to the causative pathogen, as evidenced by the formation of an induration on the skin at the site of injection [2]. As such, individuals who have received the Bacille Calmette-Guérin (BCG) vaccine often have a false-positive TST result, thus complicating the interpretation of the TST, particularly in children [13]. IGRAs on the other hand, which test for cell-mediated immunity via in vitro blood testing, use antigens more specific to TB than those used in the TST, thus increasing their specificity for detecting LTBI compared to the TST, as well as allowing the differentiation between BCG-related immunity and actual LTBI [2].

The purpose of this review is to synthesize the evidence regarding effective screening procedures for paediatric LTBI in areas epidemiologically similar to the Canadian Indigenous community setting (high-risk populations within low-burden countries) in order to optimize the current targeted screening strategy for paediatric LTBI in this population. As per the WHO’s definition, low-burden countries are those with less than 100 reported TB cases annually per 1 million people in the population [14]. As several components and considerations inform the overall screening strategy suited to a particular epidemiological context, the specific research questions investigated in this review are outlined below:

1. What overall approaches to screening for LTBI are used among high-risk groups in low-burden countries? If a targeted rather than universal screening strategy is applied, which subpopulations are targeted (e.g. school-age children from high-incidence countries)?
2. Which screening tools are used in high-risk groups in low-burden countries, and what is their specificity and sensitivity in paediatric populations in these settings?
3. Which risk factors inform targeted screening (e.g. community-level incidence, or individual risk factors)?

## Methods

The methodology and results of this systematic review are reported according to PRISMA guidelines [15]. A search of the Medline and Embase databases was conducted on the OVID platform, using the search term: (latent tuberculosis OR latent tuberculosis.tw,kf.) AND (exp Mass Screening OR screening.tw,kf.) AND (adolescent OR child* OR pediatric OR paediatric OR youth), substituting MeSH terms specific to each database, with no limits placed on language or date of publication. Studies that met the eligibility criteria of discussing strategies or tools for LTBI screening in paediatric populations, published in any language, were retained. Studies pertaining to LTBI screening in a Canadian Indigenous setting were also included, even if they did not specifically focus on paediatric subjects.

**Fig. 1:**
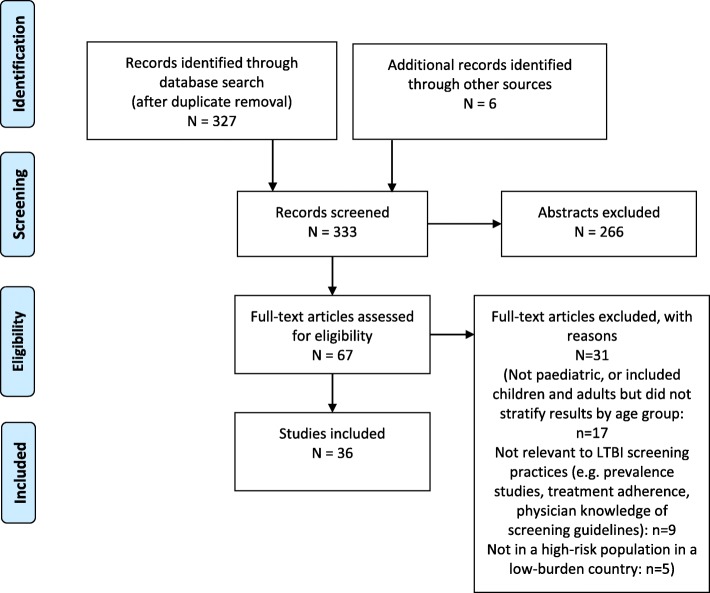
Study Screening and Exclusion Flowchart (PRISMA, [15])

Studies that did not focus on high-incidence communities within an otherwise low-burden country were excluded, in order to ensure the identification of screening methods applicable to and feasible in the Canadian Indigenous context. Studies not relevant to screening strategies, for example, those that evaluated treatment adherence or physician compliance with screening protocols were also excluded, as were studies that compared screening tools but did not report results specific to the paediatric subgroup of their sample. Further eligible studies were identified by manual searching, and grey literature sources such as reports by governmental and non-governmental organizations were also consulted. A diagram of the screening strategy is shown in Fig. 1.

As this was a synthesis of pre-existing literature, ethical approval was not required.

Data was extracted from included studies by a single reviewer, using a data extraction form created in Microsoft Excel. The methodological quality of included primary research studies (*n* = 25) was assessed using quality assessment tools appropriate to different study designs. The quality of cohort or cross-sectional studies (*n* = 9) (in this case those evaluating an LTBI screening strategy) was assessed via the National Heart, Lung and Blood Institute’s Quality Assessment Tool for Observational Cohort and Cross-Sectional Studies, [16] whilst the quality of studies evaluating screening tools for LTBI (*n* = 15) was evaluated based on items from the Quality Assessment of Diagnostic Accuracy Studies checklist [17]. The quality of modelling studies (*n* = 1) was assessed based on the ISPOR Principles of Good Practice for Decision Analytic Modelling in Health-Care Evaluation [18]. Risk of bias across studies is considered in terms of both publication bias as well as selective reporting in individual studies, the latter of which is captured as a criterion in the aforementioned tools. The resulting study quality scores were taken into account when drawing conclusions regarding potentially effective LTBI screening strategies for the Canadian Indigenous context.

As outlined by the research questions above, the results of the review will be discussed with respect to both, the evaluation of screening programs or strategies, and the comparison of screening tools in Indigenous and other high-risk paediatric populations. Regarding the optimization of screening strategies, particular attention will also be paid to whether the presence of specific risk factors informs the targeting of screening among certain groups, and whether consideration of these risk factors as a basis for targeted screening is applicable to the Canadian Indigenous setting. Recommendations for the optimization of screening in the Northern Ontario Indigenous communities will then be provided based on the findings of studies conducted in populations with similar incidence rates.

## Results

### Literature search

The initial literature search returned 327 studies after duplicate removal, 266 of which were excluded after abstract screening, due to not reporting on paediatric populations (*n* = 40), not discussing TB screening practices (*n* = 194), reporting on active rather than latent tuberculosis (*n* = 1), not taking place in a high-prevalence population within a low-burden country or a community otherwise comparable to Canadian Indigenous populations (*n* = 16), or the full article being unavailable (*n* = 15). Of the 61 studies remaining for full article review, 31 were excluded due to: not focusing on a paediatric population, or including both adults and children but not stratifying results by age groups (*n* = 17), not providing information relevant to screening practices (*n* = 9, for example, prevalence or treatment adherence studies), and not pertaining to a high-risk community within a low-burden country (*n* = 5), leaving 30 eligible studies. Further manual searches identified 6 additional relevant studies, resulting in a total of 36 studies being included in the review (original studies: *n* = 25, review papers or policy recommendations: *n* = 11). Apart from the 36 articles in academic journals, government documents such as the Canadian Tuberculosis Standards [5, 19, 20] and the Australian National Guidelines for the Management of Tuberculosis [21] were also consulted, as were reports by non-governmental organizations such as the World Health Organization [1, 3, 22, 23].

Of the 25 original studies, seven were conducted in Canada, with four evaluating a screening program (*n* = 1) or tool (*n* = 3) in Canadian Indigenous communities, [24–27] (two of which were not specifically in children) [24, 25], one evaluating a school-based screening program that included both Indigenous and non-Indigenous children, [28] and two further studies in non-Indigenous children [29, 30].

Overall, 10 studies evaluated a screening program or strategy in a Canadian Indigenous [24] or high-risk paediatric population in a low-burden setting, [24, 28, 30–37] whilst 13 others evaluated the effectiveness of IGRAs [25, 26, 29, 38–47] in a paediatric population, one investigated the effect of BCG vaccination on TST-positivity, [27] and one the effect of age on IGRA positivity [48]. Relevant characteristics of the included studies that investigated screening strategies are summarized in Table 1, whilst those evaluating screening tools are summarized in Table 2. A summary of the screening strategies evaluated in the included studies, listed by regional or community-specific incidence rates as retrieved from the scientific literature or governmental sources, is provided in Table 3. In the case of studies conducting or recommending risk-based screening strategies, the specific risk factors considered in the prioritization of targeted screening are outlined in Table 4.

**Table 1:** Studies investigating effective screening strategies for paediatric LTBI in high-risk populations within low-burden countries (by study population)

First Author (Reference)	Year	Study setting	Study population	Sample size	Study objective	Screening program/strategy	Screening tool used	Findings/Recommendations regarding screening strategy
Indigenous Communities								
Alvarez [24]	2014	Nunavut, Canada	A high-risk Indigenous community in Iqaluit, Nunavut	444	To evaluate a door-to-door LTBI screening strategy in a Canadian Indigenous community	Door-to-door screening, with targeting of dwellings screened based on location within a high-incidence area (> 5 cases in the last 5 years)	TST	• Screening based on high-risk location (rather than individual factors) was effective in this setting • 42 previously unidentified cases were identified (34% of the total incidence in the area at the time). • These cases would not have been identified via the current conventional screening practices
Pre-kindergarten or school-aged children								
Flaherman [31]	2007	California, USA	Pre-kindergarten children in California	NA^a^	To evaluate the cost-effectiveness of universal vs. targeted screening for paediatric LTBI	Compared universal screening for paediatric LTBI prior to kindergarten entry to targeted screening based on the presence of at least one risk factor for LTBI	TST	• Universal screening had a higher incremental cost compared to targeted screening per prevented case. • Targeted screening would result in 1.89 missed TB cases per year (in areas with at least 252,405 children aged 5 among which TST testing is conducted)
Gounder [32]	2003	New York, USA	School-age children in New York receiving a TST between 1991 and 1998	788,283	To assess adherence and utility of a change in paediatric LTBI screening policy	Universal screening of new entrants to primary and secondary school replaced by screening only in secondary school entrants	TST	• More targeted screening among high-risk secondary school children would be more cost-effective (higher likelihood of identifying LTBI cases)
Yuan [28]	1995	Toronto, Canada	High-risk elementary and secondary school students in Toronto, Canada	720	To evaluate a school-based screening program in Toronto, Canada	Targeted screening based on risk, Indigenous children and children born in a country of high TB endemicity were selectively screened via TST (> 10 mm considered positive)	TST	• Poor participation (40.6%) resulted in the fact that the program prevented only 3 potential TB cases, therefore not cost-effective (cost to prevent > cost to treat, although this should be considered with caution, given that indirect costs of TB (such as QALYs), and the costs of treating secondary cases were not included in the cost-effectiveness analysis). • However, may be more cost-efficient in higher-incidence communities
Taylor [37]	2008	Newcastle, UK	Children who had a QFT-G performed at Newcastle general hospital	120	To evaluate the effect of the NICE guidelines on paediatric TB screening practices	NICE guidelines mandate the follow-up of TST+ patients with a QFT-G or T-SPOT to determine further action. Due to the lack of data on the sensitivity of IFNy assays in children, this may identify fewer cases than with the use of the TST alone for decision-making regarding possible LTBI cases	TST and QFT-GIT	• 85% fewer would have received prophylaxis under the NICE guidelines (compared to prior to implementation of the guidelines) • 2% of possible active TB cases would not have been identified. • TB management based on IGRAs is more economical in low-burden settings, although it may also be associated with lower case identification
Minodier [30]	2010	Montreal, Canada	Immigrant school children and their classmates in Montreal, Canada	4375 (3401 tests read)	To evaluate a school-based LTBI screening and treatment program for immigrant children in Canada	Children (10–12 years old) in classes with a high proportion of immigrants were targeted for screening by TST	TST	• Program cost-effectiveness and case identification could have been improved by targeting at-risk children, rather than at-risk classroom groups • Overall 777 (22.8%) TST+ (≥10 mm) • More specific case selection/targeting would be beneficial in low-burden settings • Advocates the use of a risk factor questionnaire for more targeted screening.
Immigrants (including internationally adopted children)								
Panchal [33]	2014	Leicester, UK	Recent immigrants to Leicester, UK	59,007 (10,515 children < 16 yrs)	To evaluate the effectiveness of LTBI screening after first primary care registration of recent immigrants (11-year retrospective study)	Targeted screening of immigrants recently registered with primary care services	Not mentioned	• 31.2% (15/48) of TB cases could have been prevented through screening in < 16 yrs. ^b^ at the time of first primary care registration after immigration. • Using first primary care registration as a flag for targeted screening of immigrants is effective in a high-burden community within a low-burden country.
Pareek [35]	2011	UK	177 Primary care facilities in the UK	177 (primary care centres)	To evaluate the different screening methods used to screen immigrants for LTBI in primary care facilities throughout the UK	Screening of recent immigrants registering at primary care centres (methods of screening varied across centres)	TST and IGRA	• Only 107/177 (60.4%) of primary care facilities screened for LTBI. • Primary care centres in high-risk areas were less likely to screen immigrants (35.0% vs. 68.1%, *p* < 0.0001) • More targeted and evidence-based screening policies needed. • Of those that did screen for LTBI, factors for targeting screening included: < 16 yrs. from countries with a TB incidence > 40/100,000, anyone from countries with a TB incidence > 500/100,000, or immigrants from Sub-Saharan Africa
Pareek [34]	2011b	Lancashire, Yorkshire and London, UK	Immigrants attending healthcare centres in the UK	1229 (< 16 yrs., *n* = 36)	To evaluate the cost-effectiveness of targeting LTBI screening in immigrants based on age group and TB incidence in country of origin	Various screening methods for immigrants evaluated based on incidence in country of origin	QFT-GIT	Most cost-effective screening strategy: screening those < 16 yrs. from any country with TB incidence > 40/100,000 (and > 250/100,000 for 16–35 yrs)
Trehan [36]	2008	USA	Internationally adopted children in the US (who had a TST within 2 months of arrival)	527 (191 repeat-tested)	To investigate whether repeat testing of internationally adopted children increases LTBI case identification	Repeat testing (via TST) of internationally adopted children 3 months or more after arrival (with the initial test having been taken within 2 months of arrival)	TST	• 31/191 (17.7%) of those with an initially negative TST had a positive follow-up TST. • Having a positive follow-up TST was associated with malnourishment • Repeat TST testing in vulnerable groups may be warranted to identify further cases

^a^Not applicable, cost-effectiveness and clinical decision analysis ^b^ yrs. = years old QFT-GIT = Quantiferon Gold In-Tube

**Table 2:** Studies investigating the feasibility and performance of screening tools for paediatric LTBI (by study population)

First Author (Reference)	Year	Study setting	Study population	Sample size	Study objective	Screening tool used	Comparator tool	Comparative screening results (SP, SE, discordance)	Findings/Recommendations regarding screening tools
Indigenous Communities									
Alvarez [25]	2014	Nunavut, Canada	A high-risk Indigenous community in Iqaluit, Nunavut	256 (with both TST and IGRA results)	To evaluate the feasibility of the use of IGRAs for LTBI screening in the Nunavut Indigenous population	IGRA	TST	44/256 (17.2%) discordant results, most of which occurred in people with multiple BCG vaccinations or those who were vaccinated after infancy	• 18% IGRAs positive, 32% TSTs positive. • IGRAs are a valid screening tool for LTBI in Nunavut, as most of the community is BCG-vaccinated, making IGRAs more specific.
Kwong [26]	2016	Sioux Lookout, Ontario, Canada	Indigenous adolescents in northern Ontario (screened at age 14)	11	To evaluate the IGRA for LTBI screening in a Canadian Indigenous community	IGRA	TST	7/11 had a positive TST, of these 7, all had a negative IGRA and none developed symptoms of active TB disease.	• Recommends use of IGRAsv due to high proportion of false-positive TST in BCG vaccinated adolescents.
Reid [27]	2007	Saskatchewan, Canada	Preschool children (0–4 yrs) living in Indigenous reserve communities in Saskatchewan	2953 (1086 BCG+, 1867 BCG-)	To investigate the effect of BCG vaccination on TST results in Canadian Indigenous children	TST	None		• More positive TSTs among BCG+ children at 5 mm for 0–4 yrs., but no longer significant in 3–4 yrs. at > 10 mm • Need to take BCG vaccination status and age into consideration when using TST to screen for LTBI in Indigenous communities
Immigrant or refugee children									
Howley [41]	2015	USA	Children (2–14 yrs) immigrating to the US from Vietnam, the Philippines or Mexico	2520	To evaluate the QFT vs TST for LTBI screening in immigrant children	QFT	TST	• kappa = 0.20. QFT displayed stronger association with presence of risk factors for LTBI	• Recommends the use of QFT in immigrant children (2 yrs. and above)
Losi [42]	2011	Modena, Italy	Immigrant children and adolescents	621	To evaluate the QFT-GIT as a screening tool for LTBI in children immigrating to Italy	IGRA (QFT-GIT)	TST	• 104/621 TST+ • 80/621 QFT+ • 50 TST+/QFT+, • 30 TST inconcl./QFT+ 4 active TB cases suspected, all QFT-GIT+/TST+	• QFTs useful for screening in low-burden settings, however, due to continued lack of research regarding sensitivity of IGRAs in younger children, TST positivity should not be disregarded
Lucas [43]	2010	Perth, Australia	African and Burmese refugee children resettling in Australia	524	To evaluate the QFT-GIT and T-SPOT.TB as screening tools for LTBI in refugee children resettling to Australia	QFT-GIT and TB-SPOT.TB	TST	• QFT-GIT and TB-SPOT.TB showed high concordance (k = 0.78, *p* < 0.0001). • Both had poor concordance with TST: 50%. • High failure rates (14 and 15% respectively) of T-SPOT and QFT-GIT	• High proportion of inconclusive results for both IGRAs suggests TST remains useful alternative.
Salinas [47]	2015	Spain	Undocumented immigrant children (< 19 yrs) in Basque Country, Spain	845	To determine the prevalence of LTBI in undocumented immigrant teenagers using QFT	QFT-GIT	TST	63% overall, 57% positive and 96% negative concordance.	• Screening high-risk subgroups of the population in low-incidence countries is recommended. • The use of QFT as opposed to TST in this group reduced the provision of preventive treatment by 43%
Children considered at risk based on suspected TB exposure/contact with a TB case (among other risk factors, including immigration or adoption)									
Bergamini [56]	2009	Italy	At-risk children (contacts of TB cases and recent immigrants) (0–19 yrs)	496	To investigate the effect of age on IGRA effectiveness	IGRAs (QFT-GIT, QFT-G, T-SPOT.TB)	None	• TST: uncertainty of accuracy in BCG-vaccinated children. QFTs: more indeterminate results in those < 4 yrs. compared to older children • T-SPOT: lower proportion of indeterminate results compared to QFTs	Need to take into account age and BCG vaccination status when using the TST for LTBI screening
Connell [38]	2006	Melbourne, Australia	High-risk children (suspected contact with an active case, recent arrival from high-incidence country, clinical suspicion)	106	To evaluate the effectiveness of LTBI screening with IFN-y vs. TST	IFN-γ assay	TST	• IFN-/TST+ in 70% of TST+ cases • Kappa = 0.3	TST recommended for screening among high-risk children, as TST positivity was a better predictor of the possibility of actual LTBI (those with household contacts with TB were more likely to be positive by TST than by IFN-γ assay, although this does not necessarily translate into better predictiveness of the TST given that not all household contacts will necessarily develop LTBI).
Grare [39]	2010	Nancy, France	Children considered at risk for TB due to clinical suspicion, an adult case contact or recent immigration from an endemic region (< 18 yrs)	51 (44 with test results)	To evaluate the effectiveness of QFT-GIT vs. the TST for the identification of paediatric LTBI	QFT-GIT	TST	84% agreement between the two tests	• TST remains a useful predictor of LTBI. • High levels of inconclusive IGRA results suggest more studies are needed to determine its increased effectiveness vs. TST
Sali [45]	2015	Italy	Children (0–14 yrs) with suspected active TB, exposure to an adult case, or healthy adopted children	621	To evaluate the QFT-GIT for paediatric active TB diagnosis and LTBI screening	QFT-GIT	None		• Usefulness of IGRAs in younger children: 0–12 month age group more likely to have indeterminate QFT results (*p* = 0.001). • Children < 8 months: impaired ability to respond to mitogen compared to those > 8 months (but not statistically significant)
Salinas [46]	2011	Spain	Children < 17 yrs. in Basque Country, Spain, that had previous TB contacts	160	To compare the QuantiFERON-TB gold in-tube test to the TST for the detection of LTBI in children with TB contacts	QFT-GIT	TST	95–96% concordance (100% in non-vaccinated children and children < 5 yrs)	• QFT-GT reduced preventive treatments by 28–34% and is therefore recommended in low-incidence countries
Other Paediatric Populations									
Grinsdale [40]	2016	San Francisco, USA	Children < 15 yrs. screened for TB at 20 community clinics in San Francisco	1092	To assess the concordance of QFTs and the TST in a low-burden setting	QFT-GIT	TST	• 79% discordance (TST+/IGRA-) in BCG-vaccinated foreign-born children vs. 37% discordance in non-vaccinated US-born children. • Children > 5 yrs. also significantly more likely to have discordant results	• QFT vs. TST discordance was high, however, QFT has high NPV, as no TST+/QFT- children developed active TB disease in the 5 years of follow-up. • QFT may be a better predictor of risk of progression to active disease in low-burden setting
Mekaini [44]	2014	Abu Dhabi, UAE	Children (1–19 yrs) attending health centres in Abu Dhabi for routine care	699 (669 gave blood sampling consent)	To evaluate the QFT-GIT for paediatric LTBI screening	QFT-GIT	Risk factor questionnaire	• QFT positivity was low (4/669, 0.6%), however it identified two LTBI cases that would have been suspected negative based on the risk factor questionnaire alone	QFT is recommended, depending on the estimated prevalence of TB in the population
Rose [29]	2014	Toronto, Canada	Paediatric HIV patients (< 19 yrs)	81	To evaluate the QFT-GIT for LTBI screening in HIV-positive children	QFT-GIT	TST	96% TST-/QFT- concordance, but TST+/QFT+ concordance was low.	Use of QFT in HIV-positive children is valid, although low correlation with risk factor assessment (5 mm cut off used for the TST)

**Table 3:** Screening recommendations in low-incidence countries based on TB incidence, positive predictive value of IGRAs and TST for progression to active TB disease, and number needed to screen

Country or Region	Risk Sub-group Studied	Screening Tool	2015 National TB Incidence (reported cases/million population/year) [58]	PPV ^a^ for Progression to Active Disease	NNT ^b^ or NNS ^c^	Screening Tool Recommendations	Screening Strategy Recommendations
Canada	Indigenous individuals [24] ^d^	TST	45.55	NR	NNS: 5.3	Use of the TST in the paediatric population, although IGRAs may be preferable in areas where BCG vaccination persists [26, 27]	Location-based screening in Indigenous communities with > 5 TB cases in the past 5 years [24]
	Individuals in long-term care facilities^d^ [59]	TST		NR	1410		
Australia	Immigrant/refugee children or child contacts of TB cases	TST & IGRAs	52.25	NR	NR	Although prior studies indicate potentially poorer sensitivity of IGRAs compared to the TST, [38, 43]the basis of this conclusion in the absence of a gold standard diagnostic tool is unclear, and a more recent study suggests poorer sensitivity of the TST compared to IGRAs for the detection of LTBI, based on a prospective follow-up of study subjects. [44]	
Italy	Immigrant children and child contacts of TB cases	TST & IGRAs	62.82	NR	NR	Use of IGRAs recommended in BCG-vaccinated paediatric populations [56]	
Spain	Immigrant children [47]	QFT-GIT & TST	91.12	NR	NR	Use of IGRAs is preferential in low-incidence settings to prevent unnecessary prophylactic treatment	Risk-based screening: contacts of TB cases and undocumented immigrant children [46, 47]
	Contacts of TB cases [46]	QFT-GIT & TST		NR	NR		
United Kingdom [33]	Immigrant children from low-incidence countries (< 150/100,000)	NR	96.00	NR	NNS: 5291	IGRAs are more cost-effective in low-incidence countries [37]	Risk-factor-based screening: immigrants from high-incidence countries [33–35]
	Immigrant children from high-incidence countries (> 500/100,000)	NR		NR	88		
United States	Immigrants ^d^ [60]	TST	29.66	NR	NNS: 150	IGRAs are a better predictor of progression to active disease in paediatric populations [40, 41]	Risk-factor-based screening: immigration status, malnutrition [31, 32, 36]
Europe ^d^ [54]	Immune-compromised individuals [61]	TST QFT-GIT T-SPOT.TB	NA	1.5 0.9 1.3	NNT: 50 80 60		Targeted screening of close contacts [54]
	Contacts of TB cases [62]	QFT-GIT T-SPOT.TB	NA	1.9 0.7	37 37		

^a^PPV = Positive Predictive Value (in this case, the number of individuals progressing to TB disease among those with a positive IGRA result)

^b^NNT = Number Needed to Treat (in this case, the difference between the number of TB cases developing among individuals testing positive via IGRA in those receiving compared to not receiving prophylactic treatment)

^c^NNS = Number Needed to Screen (the number of individuals needed to screen in order to prevent one TB case)

^d^Not in exclusively paediatric populations

*NA* Not applicable

*NR* Not reported

**Table 4:** Risk factors considered by included studies for prioritization of risk-based targeted screening in paediatric populations

Author (Reference)	Year	Study Setting	Study Population	Risk Factors Considered for Risk-based Screening	Definition or Measurement of Risk Factors	Implications for Risk-based Screening in the Canadian Indigenous Population
Population/Community-Level Risk, Incidence-Based Risk						
Alvarez [24]	2014	Nunavut, Canada	A high-risk Indigenous community in Iqaluit, Nunavut	Community-level high TB incidence	Communities with > 5 TB cases in the past 5 years considered high-incidence	Community-level incidence rates may be an effective guide for prioritizing screening in Canadian Indigenous communities.
Minodier [30]	2010	Montreal, Canada	Immigrant children and their classmates in Montreal, Canada	Immigrant status, or being in the same class as a child with immigrant status	NA	The study’s conclusion that case identification would have been improved by targeting only immigrant children, rather than screening entire classes that contain immigrant children suggests that increased consideration should be given to the actual incidence rates underlying the classification of certain groups as high-risk, rather than basing screening solely on membership of or contact with these groups themselves.
Yuan [28]	1995	Toronto, Canada	High-risk (indigenous or immigrant) school children	Birth in a TB-endemic country Being Indigenous	NA	Similar to Minodier et al.’s findings above (2010), the fact that this strategy was found to be cost-ineffective suggests the importance of incidence-based screening in specific communities, rather than screening entire demographic groups based on status, i.e. Indigenous/immigrant.
Panchal [33]	2014	Leicester, UK	Recent immigrants to Leicester, UK	Immigrant status	Immigrants identified upon first registering for primary care	Number needed to screen (NNS) was lowest in certain strata of the immigrant population, according to age and TB incidence in their country of origin (specifically 16–35 year olds from areas with TB incidence ranging from 150 to 499/100000), highlighting again the relevance of incidence-based screening in the case of potentially high-risk sub-populations in low-burden countries.
Pareek [35]	2011a	UK	Immigrants attending 177 Primary care facilities in the UK	Sub-factors for risk among immigrants: - Age - TB incidence in country of origin - Geographic region of origin	TB incidence > 40/100,000 in country of origin among < 16 year-olds TB incidence > 500/100,000 in country of origin, regardless of age Immigrating from Sub-Saharan Africa	See above
Pareek [34]	2011b	Lancashire, Yorkshire and London, UK	Immigrants attending healthcare centres in the UK	Sub-factors for risk among immigrants: - Age - TB incidence in country of origin	TB incidence > 40/100,000 in country of origin among < 16 year-olds TB incidence > 250/100,000 in country of origin among 16–35 year-olds	See above
Individual Risk						
Trehan [36]	2008	USA	Internationally adopted children in the US	International adoption	NA	The rationale for screening in this population is again related to the high TB incidence rates in certain countries of origin, highlighting the utility of actual incidence-based screening.
				Malnourishment	Weight-for-age *z* score <−2.0	Although malnourishment was not a factor considered for initial targeted screening in this study, the finding that malnourished children are more likely to have a positive TST upon repeat testing indicates the potential relevance of including this risk factor in targeted screening efforts among Canadian Indigenous children, as Indigenous communities in Canada are characterised by a high prevalence of food insecurity. (It should be noted that the possibility of boosting of TST reactivity upon repeat testing is acknowledged in the study, but that it is considered less likely to have had an effect in the study, as repeat testing was conducted 3 months after initial testing).
Flaherman [31]	2007	California, USA	Pre-kindergarten children in California	High-risk children based on risk factor questionnaire	The presence of one or more risk factors included in the Paediatric Tuberculosis Collaborative Group Risk Factor Questionnaire [63] ^a^	As the study found risk-based screening more cost-effective, the use of a risk factor questionnaire to guide targeted screening may be warranted in the Canadian Indigenous population, if adapted based on risk factors relevant to the Indigenous context, such as poor housing conditions, food insecurity and relevant co-morbidities (See ^b^).
Bergamini	2009	Various (see Table 2)		Regular contact with an active TB case (e.g. having a household member with TB)	NA	Although these studies evaluated the accuracy of screening tools rather than a risk-based screening strategy, close contact with an active TB case is a relevant risk factor to consider for targeted screening in paediatric Indigenous communities, due to the comparatively high prevalence of TB in this population in comparison to the overall Canadian population.
Connell	2006					
Grare	2010					
Grinsdale	2016					
Sali	2015					
Salinas	2011					
Salinas	2015					
Alvarez [5] (Canadian TB Standards) ^b^	2014	Canada	Canadian Indigenous people	Renal disease Diabetes HIV co-infection Malnourishment Inadequate housing	NA	The Indigenous population experiences a higher prevalence of these risk factors for TB infection than the general Canadian population, underlining their potential relevance in risk-based screening in Indigenous communities.

^a^This risk factor questionnaire was initially developed for use in the U. S. and includes the following factors:

1) Birth of the child outside the U. S.

2) Travel outside the U. S.

3) Exposure to an active TB case

4) Close contact with an individual who has had a positive TST

5) Contact with anyone who has been in jail or a shelter, uses illegal drugs, or is HIV-positive

6) Consumption of unpasteurized dairy products

7) Birth of a household member outside the U. S.

8) Travel of a household member outside the U. S.

Administering a TST was recommended in the original study in the case of the presence of > 1 of the above factors. [63]

### General considerations for targeted LTBI screening of high-risk groups in low-burden settings

Considering the need to optimize LTBI screening in a manner that minimizes unnecessary screening and costs whilst still allowing a sufficiently high likelihood of case identification, a study conducted in California evaluated the cost-effectiveness of universal screening for paediatric LTBI prior to kindergarten entry compared to targeted screening based on the presence of at least one risk factor for LTBI (identified via the Paediatric Tuberculosis Collaborative Group risk factor questionnaire). The study reports that targeted screening was more cost-effective than universal screening in this low-prevalence setting, with the use of universal screening instead of targeted screening resulting in an incremental cost of over 100,000 USD per prevented TB case, with each year of the use of targeted screening resulting in only 1.89 missed cases over the course of the next 20 years (based on a scenario in which at least 252,405 children aged 5 live in areas where TST testing is conducted) [31]. Furthermore, a recent study in a similar low-burden setting (San Francisco, USA) conducted a retrospective analysis of the results of routine community-based paediatric screening for LTBI (using the QFT-GIT IGRA) finding QFT-GITs to be positive in only 6.6% (72 of 1092) of children tested, underlining the practicality of more specifically targeted screening in low-burden settings [40]. This study was found to be of good methodological quality (meeting 5/6 criteria on the Quality of Diagnostic Accuracy Studies checklist), having a sample representative of the general population of interest, and sufficiently reporting study limitations and uninterpretable test results (see quality assessment results section, Fig. 3, and Appendix Table 6). Of course however, it is important to note that, given the absence of an established gold standard diagnostic test for LTBI, conclusions regarding the reliability and validity of the results, and in turn the value of the study as an evidence base, are limited. Within the limits of the strength of its evidence, this study therefore suggests that in light of potential resource and cost constraints, a targeted approach to LTBI screening among particular risk groups is favourable in an otherwise low-burden setting such as Canada. However, the factors on which to base this targeted approach among Canadian Indigenous children should be explored in the specific context of the epidemiological realities of the disease in this population.

Given the finding in the above studies that support targeted screening of risk groups to be more cost-effective than universal screening in low-burden countries, it is relevant to determine which risk factors need to be considered when identifying target groups for targeted screening. In this regard, the included studies indicate that considerations for the targeting of screening programs of specific at-risk subpopulations include both community-level and individual-level risk factors. For example, a study focusing on individual-level risk factors for paediatric LTBI highlights the importance of environmental as well as host-specific factors in the development of LTBI, firstly underlining exposure to an active TB case as a significant environmental risk factor. More precisely, it emphasizes that the risk associated with exposure to active cases is influenced by both the number of cases and the duration of exposure [49]. Importantly, the study also identifies the confined nature of the site of exposure as a significant predictor of an increased likelihood of infection, [49] which is particularly relevant to the Canadian Indigenous context, as over-crowded and inadequate housing conditions are a continued concern in Indigenous communities across Canada [5].

Further individual risk factors include immunodeficiency, diabetes and poor kidney function, [49] which are also pertinent factors to consider as priorities for targeted screening in Indigenous communities, as, for example, diabetes mellitus occurs at a higher prevalence among Indigenous people than in the general Canadian population [5]. In addition, it is noteworthy that these factors do not only influence the likelihood of infection, but also the risk of progression to active disease, [49] making it all the more important to screen for latent infections among these risk groups. In summary, the considerations of exposure, congregate living conditions and relevant comorbidities may therefore be significant individual-level risk factors to consider in the targeted screening of paediatric LTBI among the Canadian Indigenous population.

In contrast to the use of individual-level risk factors as the focus of targeted screening strategies however, location-based screening, or in other words, screening in a community based on the fact that its TB incidence rate is significantly higher than in the general population, may also represent an efficient method of LTBI screening in high-risk communities. There is however little consensus in the existing literature regarding an approximate cut-off for what constitutes this significantly higher incidence rate at which location-based screening is warranted [50]. Nonetheless, this location-based or community-based approach to screening has been shown to be effective in the Canadian context, as demonstrated by the increased identification of LTBIs resulting from a location-based screening program implemented in Nunavut, [24] suggesting that location-based risk rather than individual risk is a pertinent consideration for the basis of targeted screening in Canadian Indigenous settings. The recommendations of the included studies evaluating targeted screening strategies and programs are summarized in Table 1, and these recommendations are stratified by local incidence rates in Table 3.

### Targeted screening strategies in high-risk paediatric populations in low-burden countries

As immigrants from TB-endemic to low-burden countries also represent high-risk populations for TB infection, screening procedures for immigrant children to low-burden countries may to some extent be relevant to the consideration of screening strategies for the Canadian Indigenous population as well. In Canada, a TST or IGRA is recommended only for high-risk immigrant children, however, a review on paediatric LTBI screening among immigrants to low-burden countries indicates high heterogeneity among countries with respect to screening strategies [51].

**Fig. 2:**
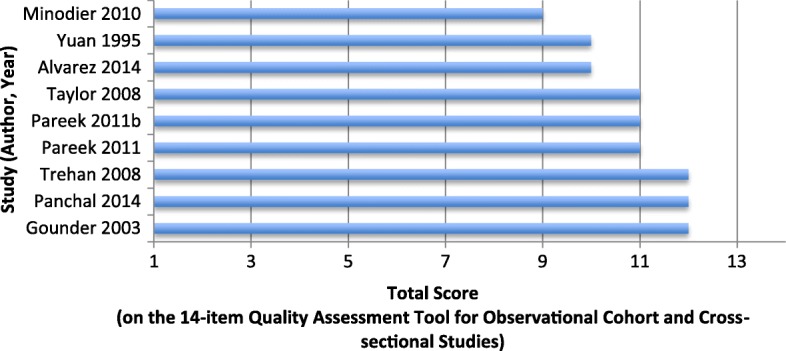
Quality assessment of cohort and cross-sectional studies

An evaluation of a school-based TB screening program conducted in Toronto, Canada, between 1992 and 1993 involved the targeted screening (using the TST) of Indigenous children as well as immigrant children born in TB-endemic countries and attending Toronto schools [28]. In total, 720 children were screened, and 22.5% of these had a positive TST result (using a wheal size of 10 mm). Despite this identification of a considerable number of LTBIs, the screening program was concluded to be relatively ineffective, firstly due to low participation, as only 40.6% of the 1775 eligible students were ultimately screened, including only one out of 22 eligible Indigenous students (this student had a negative TST). A further limiting factor of the program’s effectiveness was the fact that among those that did have a positive TST result, few (44.3%) received chemoprophylaxis, meaning that screening in this case did not necessarily facilitate beneficial downstream effects in terms of increasing preventative treatment initiation and improving health outcomes. Overall, this screening strategy was estimated to have prevented only 3 potential cases, making the screening program cost-inefficient, as the distribution of the program costs over this small number of cases yielded a cost per prevented case considerably higher than the cost to treat one. Increasing the coverage of screening programs is therefore a significant factor in the potential improvement of case detection rates and the reduction of costs per prevented case [28]. It should be noted however, that from a study quality perspective, poor enrolment of the overall eligible population (< 50%) indicates inadequate sampling (according to the Quality Assessment Tool for Observational Cohort and Cross-Sectional Studies, see Fig. 2 and Appendix Table 5), and thus limits the validity of the results (more comprehensive sampling of the population of interest may have resulted in a higher number of cases prevented through the screening program, and in turn altered the study’s conclusions regarding the program’s cost-effectiveness).

Rather than increasing coverage of screening programs, other studies investigating paediatric LTBI screening in low-burden countries emphasize the need for more focus on risk factors as determinants of targeted screening strategies (see Table 4). A study conducted in Montreal, for example, evaluated a school-based LTBI screening program that involved screening whole classes that included immigrant children (rather than screening only immigrant children themselves), with a resulting TST positivity of 22.8% (at ≥10 mm). The study concluded however that the program’s cost-effectiveness and case identification could have been improved by targeting at-risk children, rather than at-risk classroom groups (as this included some children from low-burden countries), suggesting that the more specific targeting of paediatric LTBI screening is beneficial in low-burden settings [30].

Studies investigating LTBI screening among immigrants to other low-burden countries make similar recommendations regarding the need for more specifically risk-based targeting of paediatric LTBI screening in order to conserve resources as well as improve case identification rates. A study conducted in the United Kingdom (UK), for example, found that primary care centres screening for LTBI in high-risk areas (areas with high immigration rates from TB-endemic countries) were actually less likely to screen for LTBI, [35] with a further study in the UK finding that targeting screening at the paediatric subgroup of the immigrant population (specifically, children < 16 years) from high-burden countries (those with > 40/100,000 incident cases of TB) was the most cost-effective strategy for targeted LTBI screening [34]. This argues in favour of actual incidence-based targeted screening, rather than screening based solely on collective community characteristics, such as Indigenous status.

Similarly, in a study evaluating the changes in LTBI screening (via TST) in New York following policy change to discontinue mandatory screening of students entering primary schools whilst continuing screening in secondary schools revealed that screening in both age groups continued independently of the consideration of specific risk factors [32]. For example, it was found that younger children were often screened even in the absence of risk factors, whilst children in older age groups from high TB incidence countries were not necessarily screened. This was a concern considering the fact that the study also found that in comparison to US-born children, non-US-born children were more likely to have a positive TST result (proportion of TST positivity: 1.5 and 14.5% respectively), and almost 5 times more likely to have active TB disease (RR: 4.9; 95% CI: 3.5–6.8) [32], thus underlining the need for risk-based screening in a low-burden country.

Other than the need for increased consideration of risk factors in the targeting of screening for paediatric LTBI among high-risk groups in low-burden settings, the utility of repeat testing in these groups should also be explored. In a study evaluating repeat testing (via TST) of internationally adopted children in Cincinnati, U. S., 17.7% of those initially tested were found to have a positive follow-up TST. Notably, it was found that having a positive follow-up TST was associated with malnourishment (defined as a weight-for-age *z* score below − 2.0) indicating that repeat LTBI testing in vulnerable groups may be warranted [36]. As the Canadian Indigenous population experiences higher food insecurity than the general population, [5] repeat testing for paediatric LTBI may also be applicable in this context, and facilitate the identification of further LTBIs. Although the possibility of boosting of TST reactivity upon repeat testing is acknowledged in the study, it is considered less likely to have had an effect on the observed subsequent positivity in the study, as its likelihood is reduced if repeat testing is conducted more than 2 months after initial testing (as was the case in this study, in which repeat testing occurred 3 months after initial testing) [36].

### Targeted screening strategies in Indigenous populations

In an effort to consider avenues for improving TB prevention in Nunavut, Canada, a recent study evaluated the effectiveness of a door-to-door screening strategy in which the areas to be screened were determined based on previous records of high TB incidence (defined as > 5 cases over the last 5 years), rather than specific risk factors at the individual level. This approach resulted in the identification of 42 previously unidentified LTBIs, which amounts to a 34% increase in detected LTBIs in the area [24]. This suggests that focusing on specific high-incidence areas rather than certain individual risk factors may be an effective alternative strategy for comprehensively screening for LTBI in Indigenous communities.

To date, little information is available regarding screening strategies for LTBI among Indigenous populations in other low-burden countries. A study investigating TB diagnoses and risk factors among Australian children, for example, reports that 37 out of the 524 identified cases occurred in Indigenous children, (resulting in a higher annual case notification rate among Indigenous than non-Indigenous children (1.70 [95% CI: 1.20–2.34] vs. 0.56 [95% CI: 0.48–0.65] per 100,000 population)), and that active case detection through contact screening was responsible for the identification of 37% of the cases identified, [9] however, although the Australian Department of Health identifies Indigenous groups as a high-risk population for TB infection, and recommends both BCG vaccination and targeted screening in this population, a specific strategy for targeted screening in these groups is not mentioned [21]. Among the Inuit population in Greenland, a recent World Health Organization report highlights that targeted screening efforts among school children have been ineffective, based on the fact that incidence continues to rise in the region. Due to the high cost of mass screening via X-rays or IGRAs in remote locations in Greenland, the WHO recommends passive case-finding and subsequent specific location-based screening in high-risk areas [22].

### The accuracy of available screening tools for LTBI in Paediatric populations

Apart from considering effective strategies for the targeted screening of high-risk groups, determining which screening tool is most appropriate among Canadian Indigenous children is also pertinent, particularly considering the uncertainty regarding the accuracy of IGRAs in young children, [45] the reduced specificity of the TST in BCG-vaccinated children, [13] and the varying yet continued practice of BCG vaccination in some Indigenous communities [19, 52].

A review of screening methods for LTBI among high-risk groups in the United Kingdom concluded that among children, in comparison to the TST at a 5 mm cut-off, IGRAs have similar sensitivity, however, the specificity of IGRAs is higher than that of the TST at this cut-off. In contrast, the sensitivity of IGRAs was found to be higher than that of the TST at a 10 or 15 mm cut-off, whilst their specificity was lower [53]. The study therefore recommends the TST as the primary screening tool for paediatric LTBI, with an induration size of ≥5 mm as the cut-off for a positive test in children (> 5 years old) who have no record of BCG vaccination, and > 15 mm in BCG-vaccinated children, as well as an IGRA in TST-positive children [53]. It is important to note however that the review, which included both high and low TB-burden settings, found that IGRAs were only better predictors of LTBI in low-burden settings, whereas the TST remained a better predictor in high-burden settings [53]. As this difference is expected to be due to the increased presence of other factors such as malnutrition, concomitant HIV infection and other comorbidities in high TB-burden settings, these results suggest that within the Canadian Indigenous population, which is also characterised by a higher prevalence of some of these risk factors (such as malnutrition) than the general population, [5] the TST may remain an effective screening tool for LTBI in Indigenous communities.

On the other hand however, a study evaluating the use of the IFN-γ assay and the TST as screening tools in Australian children at high risk of TB reported that IGRAs were negative in 70% of children with positive TSTs, [38] and a study among a paediatric population in the USA reporting similar levels of IGRA-/TST+ discordance (79% in BCG-vaccinated or foreign-born children, 37% in US-born children) [40]. Similarly, a study conducted among a high-risk subgroup of children in France (those in contact with a suspected active case of TB, or those recently having immigrated from TB endemic countries) compared the TST to the QuantiFeron TB Gold in-tube (QFT-GIT) IGRA. This study also reported only moderate agreement between the two screening tools, with TST and IGRA positivity occurring in 27.3 and 11.4% of the 44 children, respectively [39]. Importantly, using the occurrence of suspected or confirmed cases of TB among a child’s contacts and the expected increased risk of exposure with increased age as proxies for LTBI, a study among immigrant children in the U. S. found QFT-GIT positivity to be a more accurate predictor of LTBI than TST positivity, and thus recommends IGRAs for LTBI screening among high-risk paediatric populations in low-burden settings [41].

Notably, two other studies evaluating the use of the TST in comparison to IGRAs in high-risk children (undocumented immigrant children and those with TB case contacts) in a low-burden country (Spain), highlight that the use of IGRAs avoided the provision of preventive therapy by 28–43%, indicating that IGRAs may prevent unnecessary prophylactic treatment and represent a cost-saving method of LTBI screening among high-risk groups in low-burden countries [46, 47]. This is particularly true considering that a further study found IGRAs to be a more accurate predictor of progression to active disease, as no TST+/IGRA- children developed active TB over the course of the study’s follow-up time of more than 5 years [40]. Conversely however, a recent review of the use of IGRAs in low-burden European countries concludes that a positive IGRA is not in fact an effective predictor of progression to active TB, and that its predictive value for progression to active disease is highly heterogeneous among different high-risk groups in low-burden countries [54]. Moreover, this review reported that the number needed to treat (NNT), in this case the difference between the number of TB cases developing among individuals with a positive IGRA result receiving compared to not receiving preventive treatment, was found to vary between 37 and 80 among various high-risk groups (see Table 3). The lowest NNT occurred when targeted screening was limited to close contacts of TB cases, emphasizing the potential utility of prioritizing screening in this risk group in the case of low-burden settings [54].

In the Canadian context, a study among Indigenous adolescents in northern Ontario province reports that prior BCG vaccination was associated with false-positive TST results, [26] corroborating the findings of other studies regarding the discordance between IGRAs and the TST in BCG-vaccinated children, [55, 56] and suggesting that the use of IGRAs may improve paediatric LTBI screening among the Indigenous population, particularly considering the history of neonatal BCG vaccination in northern Ontario [26]. Similarly, a previous study of paediatric LTBI among Indigenous 0 to 4 year old children found increased TST reactivity among BCG vaccinated children across this age range when an induration diameter of 5 mm was considered a positive result, however, this difference was no longer significant among the 3 to 4 year old children when an induration of > 10 mm was used as the cut-off (although it remained significant in the younger age groups) [27]. Given that BCG vaccination is administered at birth in some Indigenous communities across Canada, [19] these results suggests that within the paediatric Indigenous population, the use of the TST as a screening tool for LTBI, and its interpretation, should take into account age group and prior BCG vaccination.

As an alternative to LTBI screening via TST, a study assessing the effectiveness of the use of IGRAs for LTBI screening among an Indigenous population in Nunavut concluded that IGRAs are a valid screening tool for LTBI in this setting, as most of the community was BCG-vaccinated, making IGRAs more specific, and thereby saving resources dedicated to TB prophylaxis [25]. On the other hand however, although the consideration of prophylactic treatment on the basis of a positive IGRA rather than a positive TST is potentially cost-saving among high-risk groups in low-burden countries, several studies in paediatric populations in these settings highlight the continued scarcity of evidence concerning the accuracy of IGRAs in young children [42, 43, 45].

Conclusively, the quality of the evidence presented in these studies evaluating screening tools is limited by the absence of a gold standard diagnostic test for LTBI, leading to uncertainty regarding validity of the results. Multiple studies drew attention to the discordance between the TST and IGRAs, [26, 27, 56] and underlined that TST specificity is low (62.7–69.0%) among BCG-vaccinated children [57]. Nonetheless, several continue to advocate for the use of TST over IGRAs in young children, even in low-burden settings, due to the scarcity of evidence regarding the sensitivity of IGRAs in younger age groups, and their frequent inconclusive results in this group [39, 42, 43, 45, 56].

### Study quality assessment:

Outcomes of the methodological quality assessment for cohort and cross-sectional studies (those assessing screening strategies) (*n* = 9), assessed using the National Heart, Lung and Blood Institute’s Quality Assessment Tool for Observational Cohort and Cross-Sectional Studies [16], are shown in Fig. 2. This scale assesses studies based on appropriateness of sampling procedures, and validity and reliability of exposure and outcome measures, with a maximum score of 14 points being possible. According to this scale, the included cohort and cross-sectional studies were generally of good quality, with studies scoring on average 10.9/14 (77.8%) (range: 64.3–85.7%). All studies clearly specified their objective, clearly defined their study population, and used valid measures and timeframes for assessment of exposures and outcomes, however, some methodological weaknesses identified by the quality assessment included: a lack of reporting on whether measures were taken to blind outcome assessors to the exposure status of participants (in all studies), and in some studies, a lack of reporting on identification of and adjustment for potential confounders [30, 35, 37]. Only one study experienced a loss to follow-up greater than 20% after baseline [30]. A detailed breakdown of scores on each criterion for each study is provided in Appendix Table 5.

**Fig. 3:**
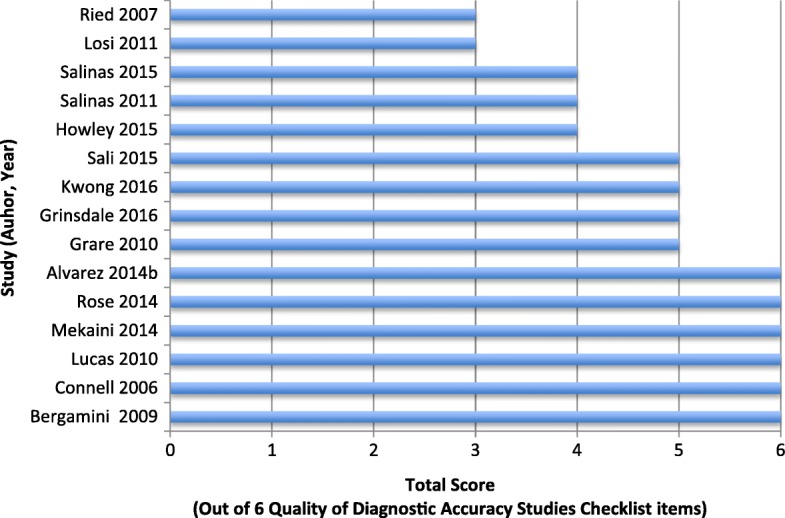
Quality assessment of diagnostics accuracy studies

The results of the quality assessment of diagnostic accuracy studies (*n* = 15) (e.g. those comparing the TST to IGRAs), assessed using 6 items from the Quality of Diagnostic Accuracy Studies checklist [17], are shown in Fig. 3. Studies scored on average 4.9/6 (82.2%) (range: 50–100%), although it should be noted that the comprehensiveness of this quality assessment is limited by the fact that questions assessing the appropriate comparison of an index test (e.g. IGRAs) to a gold standard were not included in this assessment or in the final scoring, due to the continued absence of a gold standard diagnostic test for LTBI. The true reliability of these studies may therefore be lower than what is reflected in the quality assessment. Criteria met by most studies included ensuring that the study sample is representative of the general population of interest, and clearly defining participant eligibility criteria, however, several studies lacked detail regarding how the diagnostic test was administered, [26, 27, 41] or did not report whether uninterpretable test results occurred [27, 46, 47]. In addition, reasons for participant withdrawal from the study were often not explained, so attrition biases are possible. A detailed breakdown of scores on each criterion for each study is provided in Appendix Table 6.

The modelling study of the cost-effectiveness of no screening, universal screening or targeted screening (via TST), [31] assessed based on the ISPOR Principles of Good Practice for Decision Analytic Modelling in Health-Care Evaluation, [18] was determined to be of acceptable quality, meeting most (11/18 [61.1%]) methodological and reporting criteria set out in these principles (see Appendix Table 7). Its methodological strengths included a valid model structure, which incorporated relevant inputs and outputs based on the decision-making perspective taken (health-system-level perspective), however, model validation procedures were not described, thus limiting the study’s utility as an evidence base for recommendations regarding optimal screening strategies.

Considering bias in the cumulative evidence synthesized (bias across rather than within studies), selective reporting is unlikely to have posed a high risk of bias across studies, given that, for example, all except 3 of the 15 diagnostic accuracy studies reported indeterminate or uninterpretable test results. A further potential source of bias across studies may have been publication bias, particularly given that many of the included studies are from only a few countries, and evidence from a wider variety of eligible countries may have allowed important additions or changes to the review’s conclusions regarding optimal screening strategies. However, the risk of publication bias is reduced given that no language restrictions were applied to the search, eligible studies were only those focusing on high-risk groups in an otherwise low-burden country, and relevant grey literature sources were consulted.

## Discussion

Overall**,** all studies discussing screening programs highlight the clinical and cost-ineffectiveness of universal screening programs in low-burden countries. Instead, several recommended targeted screening based on individual risk factors (such as immigrant status or exposure to an active TB case), highlighting the utility of a risk factor questionnaire as a valuable predictor of LTBI and risk of progression to active TB disease [30–32, 34, 35]. With reference to previous studies, individual risk factors relevant to the Canadian Indigenous context that should be considered in the prioritization of targeted screening include contact with an active TB case, overcrowded living conditions, and immune-compromising conditions such as HIV co-infection and malnutrition [1, 2, 49]. Other studies however recommend targeted screening based on location in a high-risk area rather than individual-level risk factors. [24, 30] Of those not screening based on individual-level risk factors, one study, conducted in a school-based setting among the general population [30] concluded that screening based on individual-level risk factors rather than on the basis of being in a high-risk area would have improved case detection, whilst the other, conducted in a Canadian Indigenous community, [24] suggests that a location-based approach allowed better case detection, perhaps suggesting that location-based screening may be the more effective strategy in an Indigenous setting, where we expect a higher overall prevalence of TB in the community. It is also important to note when considering individual risk factor-based vs. community-based screening, that Indigenous communities may find one strategy more culturally acceptable than another, and this should be taken into account.

The limitations of this review include the heterogeneity of the studies summarized, particularly in terms of the heterogeneity of the study populations with regard to BCG vaccination history and risk factors for TB infection. In addition, although the methodological quality of the included cohort and cross-sectional studies was generally high, the quality assessment of studies evaluating screening tools (e.g. the TST or IGRAs) was limited due to the lack of a gold standard for LTBI diagnosis. Moreover, the very limited reporting of number needed to screen for IGRAs and the TST in paediatric studies, or their negative predictive values in the case of BCG-vaccinated children, presents challenges for the determination of an optimal incidence-based screening strategy for LTBI in Canadian Indigenous children.

### Summary of recommendations:

Keeping in mind the aforementioned limitations, the results of this review suggest that targeted rather than universal screening is warranted in high-risk communities within low TB-incidence countries, and that the consideration of both community-level or location-based as well as individual risk factors have merit as determinants of targeted screening strategies. Although location- and incidence-based screening is likely to allow efficient case identification in the context of remote high-burden communities in an otherwise low-burden country, [24] evidence and consensus regarding a specific cut-off for high-incidence remains scarce. Moreover, as the Canadian Indigenous population is characterised by a high prevalence of co-morbidities and socio-demographic factors that predispose individuals to TB infection (such as food insecurity, inadequate or overcrowded housing, diabetes, renal disease, and HIV), it is also relevant to consider individual rather than solely location-based or community-level risk factors for targeted LTBI screening in Indigenous communities. Lastly, the choice of a context-appropriate screening tool in the case of the paediatric Indigenous population in northern Canadian communities is complicated by the history of BCG vaccination in some regions, which may result in high false positive TST readings. IGRAs may therefore represent a more accurate screening tool in this population, [26] although their accuracy in children remains contested and it should thus be kept in mind that their implementation may lead to increased missed cases. Overall therefore, a risk- or incidence-based targeted screening strategy for paediatric LTBI in a high-burden population in low-incidence countries is recommended, potentially implementing the TST as the standard screening tool, at a 5 mm cut-off for positivity, substituted by IGRAs in the communities in which BCG vaccination continues. There remains, however, a lack of evidence for the utility of screening at a specific frequency or timing, apart from during contact investigation.

## Appendix

**Table 5:** Quality Assessment of Cohort and Cross-Sectional Studies ^a^

First Author	Year	Study Design	Objective clear	Study population clearly specified	Participation rate of eligible persons at least 50%	Subjects recruited from the same or similar populations	Sample size justification, or power analysis ^b^	Exposure(s) of interest measured prior to outcome(s)	Timeframe sufficient to expect to see an association between exposure and outcome, if it exists	Levels/categories of exposure measured, where applicable ^c^	Exposure measures clearly defined	Exposure(s) assessed more than once over time	Outcome measures clearly defined	Outcome assessors blinded to exposure status of participants	Loss to follow-up after baseline 20% or less	Key potential confounding variables measured and statistically adjusted for	Total (out of 14)
Gounder	2003	RC	1	1	1	1	1	1	1	1	1	x	1	x	1	1	12
Minodier	2010	PC	1	1	1	1	x	1	1	1	1	x	1	x	x	x	9
Panchal	2014	RC	1	1	1	1	1	1	1	1	1	x	1	x	1	1	12
Pareek	2011	CS	1	1	1	1	1	1	1	1	1	x	1	x	1	x	11
Pareek	2011b	PC	1	1	1	1	x	1	1	1	1	x	1	x	1	1	11
Trehan	2008	PC	1	1	1	1	1	1	1	1	1	x	1	x	1	1	12
Alvarez	2014	PC	1	1	x	1	x	1	1	1	1	x	1	x	1	1	10
Yuan	1995	PC	1	1	x	1	x	1	1	1	1	x	1	x	1	1	10
Taylor	2008	CS	1	1	1	1	1	1	1	1	1	x	1	x	1	x	11

1 = criterion met x = criterion not met or not reported CS = Cross-Sectional RC = Retrospective Cohort PC = Prospective Cohort

^a^Based on the National Heart, Lung and Blood Institute’s Quality Assessment Tool for Observational Cohort and Cross-Sectional Studies, [16]

^b^Where studies sampled an entire population of interest (e.g. data available on all eligible individuals through health system records), a point was awarded for sample size justification)

^c^Where exposure was the absence / presence (introduction / cessation) of a particular screening strategy, “levels” of exposure are not applicable, as this exposure is dichotomous. In this case, studies were not deducted points

**Table 6:** Quality Assessment of Diagnostic Accuracy Studies^a^

First Author	Year	Sample representative of general population who will receive the test	Sample selection criteria clearly described	Execution of the index test described in sufficient detail to permit replication	Same clinical data available when test results were interpreted as would be availablewhen the test is used in practice	Uninterpretable/intermediate test results reported	Withdrawals from the study explained	Total (out of 6)^b^
Bergamini	2009	1	1	1	1	1	1	6
Connell	2006	1	1	1	1	1	1	6
Grare	2010	1	1	1	1	1	x	5
Grinsdale	2016	1	1	1	1	1	x	5
Howley	2015	1	1	x	1	1	x	4
Kwong	2016	1	1	x	1	1	1	5
Losi	2011	x	x	1	1	1	x	3
Lucas	2010	1	1	1	1	1	1	6
Mekaini	2014	1	1	1	1	1	1	6
Rose	2014	1	1	1	1	1	1	6
Sali	2015	1	1	1	1	1	x	5
Ried	2007	1	1	x	x	x	1	3
Alvarez	2014 (b)	1	1	1	1	1	1	6
Salinas	2011	1	1	1	1	x	x	4
Salinas	2015	1	1	1	1	x	x	4

1 = criterion met x = criterion not met or not reported

^a^Based on items from the Quality Assessment of Diagnostic Accuracy Studies checklist. [17]

^b^Criteria from the original checklist pertaining to comparison of the index test to a gold standard were not included, given the absence of a gold standard for LTBI diagnosis

**Table 7:** Quality Assessment of Modelling Study (Flaherman 2007 [31])

Criteria^a^	Criteria met? (1 = yes, x = no or not reported)
Structure:	
Inputs and outputs relevant to the decision-making perspective	1
Structure consistent with the theory of the disease in question	1
Structure as simple, although including essential aspects for decision-making. Simplifications, if any, justified as not significantly affecting the results.	1
Heterogeneity in the modelled population accounted for by stratifying by groups that have different outcome probabilities or costs.	1
Time horizon of the model sufficient to detect important (and clinically meaningful) differences in long-term health and cost outcomes.	1
Data:	
Data identification:	
Systematic reviews of the literature conducted on key model inputs.	x
Ranges provided in base-case estimates of all input parameters for which sensitivity analyses were done.	1
Data based on expert opinion, if used, are derived via formal methods, e.g. Delphi	x
Attempts to obtain new data prior to modeling have been considered.	x
Data modeling:	
Modeling methods follow accepted methods of biostatistics and epidemiology.	1
Data incorporation:	
Use of either probabilistic (Monte Carlo, first-order) simulation or deterministic (cohort) simulation	1
Included sensitivity analyses of key parameters.	1
Validation:	
Internal validation:	
Model subjected to internal testing through input of extreme values (or equal values for replication testing)	x
Calibration data, where available, should be from sources independent of those used to estimate inputs	x
Source code available for peer-review.	x
Between-model validation:	
Models developed independently of each other, to allow convergent validity testing	x
Significant discrepancies in model outputs compared to other published results explained	1
External and predictive validation:	
Model based on the best evidence available at the time	1
Total Score (out of 18)	11

^a^Based on the ISPOR Principles of Good Practice for Decision Analytic Modeling in Health-Care Evaluation [18]. (Since this study did not employ a transition-state model, components of the ISPOR guidelines pertaining to such models were excluded from this assessment)

## Abbreviations

BCG: Bacille Calmette-Guérin
HIV: Human Immunodeficiency Virus
IFN: Interferon
IGRA: Interferon Gamma Release Assay
LTBI: Latent TB Infection
QFT: Quantiferon
QFT-GIT: QuantiFERON TB Gold In-Tube Test
TB: Tuberculosis
TST: Tuberculin Skin Test
